# A Simulation Study on the Effect of Residual Stress on the Multi-Layer Selective Laser Melting Processes Considering Solid-State Phase Transformation

**DOI:** 10.3390/ma15207175

**Published:** 2022-10-14

**Authors:** Xiao Li, Ming Zhang, Junfeng Qi, Zhengmao Yang, Zhonghua Jiao

**Affiliations:** 1 Beijing Spacecrafts, China Academy of Space Technology, Beijing 100094, China; 2 Institute of Mechanics, Chinese Academy of Sciences, Beijing 100190, China; 3 School of Engineering Science, University of Chinese Academy of Sciences, Beijing 100049, China; 4 Dassault Systemes SIMULIA, Beijing 100702, China

**Keywords:** additive manufacturing, residual stress, laser deposition, thermo-mechanical modeling, finite element analysis

## Abstract

The selective laser melting (SLM) manufacturing process is a complex process involving moving a molten pool, rapid non-equilibrium solidification and solid phase transformation. If the thermal residual stress is too large, it may lead to warping, cracking and failure of the structures. The present work aims to establish a thermo-mechanical framework to predict temperature evolutions, molten pool configurations and residual stresses of materials in the SLM process, based on the toolpath-mesh intersection method. Moreover, the influences of the laser power, process parameters and mesh size have been discussed. The stress concentration occurred at the interface between the melt layer and substrate results in warping deformation of the materials. This work provides a novel method to reveal the resulting physical mechanism inside the molten pool in terms of residual stresses and distortions.

## 1. Introduction

Additive manufacturing is a disruptive technology for changing design paradigms and providing the way in innovative advanced structure design and applications. Among the additive manufacturing technologies, selective laser melting (SLM) possesses high laser power and small laser spot radius, and has become the most widespread, flexible, and economic method within additive manufacturing [1,2]. However, the SLM process is a multi-factor coupling process with powder, laser beam and substrates, and so on, which is a complex metallurgical process involving a moving molten pool, rapid non-equilibrium solidification and solid-phase transformation [3,4,5]. During the process, a large amount of thermal residual stresses will be generated which can affect the fracture toughness, crack growth behavior and fatigue performance of the materials [6,7,8,9]. Consequently, an accurate estimation of residual stresses and distortion is necessary to achieve dimensional accuracy and prevent premature failure of the components.

Extensive researches have been conducted to predict the temperature distribution and mechanical behaviors during the SLM process [10,11,12,13]. Loh et al. [14] proposed a model to simulate the powder-to-solid transition during the SLM process, and the effects of the laser power and scan speed on the melting dimensions and temperature change rates have been determined. Mukherjee and DebRoy [15] developed a three-dimensional model considering the transient heat transfer and fluid flow to investigate the transient temperature field during the SLM process, and they found that reducing the layer thickness can decrease the residual stresses. Roehling et al. [16] investigated the effects of temperature gradients and melting velocity on the microstructures of the 316 L stainless steel based on the ALE3D multi-physics code. Alexopoulou et al. [17] proposed a modeling method to simulate the melting pool in the SLM process, using the Volumetric Energy Density (VED) method. The research provided the basis for calculating the thermal physical parameters, and selecting the appropriate calculation method during the SLM process, so that the transient temperature field formed during the forming process could be calculated and extracted based on the numerical simulation.

With the development of SLM technology, many researchers have paid more and more attention to the stress analysis research of the SLM process [18,19]. Zhang et al. [20,21,22] developed a framework to link the processing conditions with the microstructural features, based on the previously-developed framework for the temperature and residual stress predicting. Song and Feih [23] tried to optimize the process parameters by establishing the finite element model, so that the residual stress can be reduced. Chen et al. [19] predicted the thermal residual stress distributions of the high strength tool steel during the SLM process by establishing a solid-state phase transformation model. However, due to the nonuniform heating of the laser heat source, an extreme temperature gradient will be generated, and the residual stress will occur after cooling, affecting the precision size of the products, and even making the parts warp and crack.

The goal of the present work is to establish a general framework for predicting temperature evolutions, molten pool configurations and residual stresses of Ti-6Al-4V alloys in the SLM process. To achieve that, considering the heat conduction, radiation and convection during the process, a meso-scale numerical model is proposed based on the new Plug-ins implemented in the commercial code Abaqus 2020, and the influences of laser power and process parameters on the thermal behavior of the SLM process are investigated. Furthermore, the solid-state phase transformation model is established to predict the residual stress of the SLM process, and the relationship among laser power, meshing size and residual stress is evaluated.

## 2. Numerical Models in SLM Process

It is of importance to investigate the microstructure evolution, internal defect formation, structural deformation and coupling mechanism of materials in the SLM process, so as to realize the optimization of process parameters with low cost and high efficiency. The numerical simulation method has been applied to the SLM process [24], which can intuitively solve the temperature, thermal stress, deformation and other problems involved in the additive manufacturing process through the analysis of different materials and processing parameters. In this section, a three-dimensional finite element model is established to simulate multi-layer deposition during the SLM process, which contains the heat source model, solid-state phase transformation model and the thermo-mechanical model.

Since the mechanical behaviors of the SLM process are regarded as nonlinear, the thermo-elastic-plastic theory is generally used to establish the finite element model of the SLM process. Additionally, the stress evolution in the SLM process is quite complex, so it is necessary to simplify the calculation appropriately and ensure the calculation accuracy. The following assumptions have been applied for the simulation:

(i) The vaporization and flow-induced temperature change of the liquid metal in the molten pool is ignored;
(ii) The pores or the influence of gas during processing are not considered in the model;
(iii) The creep-induced strain effects and solid-state phase transformation are neglected;
(iv) The mechanical properties and stress-strain of the material change linearly in a small time interval.
(v) Melting, solidification and particle-to-particle interactions of metal powder particles are not considered.

### 2.1. Heat Source Model

**Figure 1:**
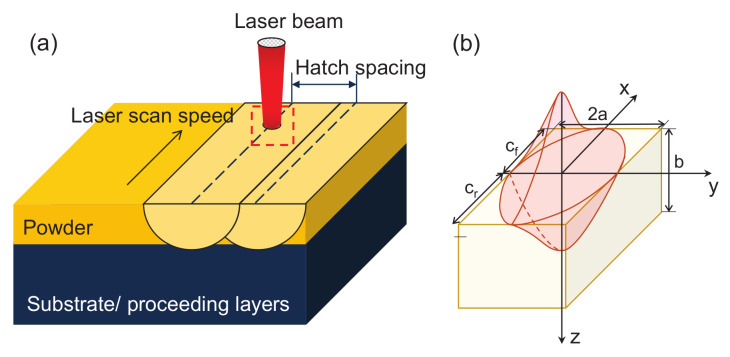
(**a**) Schematic diagram of SLM processing process; (**b**) Goldak’s double-ellipsoidal power density distribution model [26].

The heat source model has a great influence on the calculation and analysis of temperature and stress in the SLM process. In the present work, the laser heat source is considered as a distribution of volumetric heat, which can be described by Goldak’s double-ellipsoidal power density distribution model [25], and the schematic of the model is illustrated in Figure 1. Then, the volumetric heat *Q* can be expressed as,

(1) $$Q=\frac{2P\eta f_{f/r}}{abc_{f/r}\pi \sqrt{\pi }}\exp\left[-\left(\frac{x^{2}}{a^{2}}+\frac{y^{2}}{b^{2}}+\frac{z^{2}}{c_{f/r}^{2}}\right)\right]$$

where *P* is the laser power, η denotes the laser absorption rate of the material, and $v_{x}$ is the travel speed of the laser, and *a*, *b*, and $c_{f/r}$ are the morphology parameter along *x*, *y* and *z* axis of the heat source as shown in Figure 1; $f_{f/r}$ is the control parameter, and *x*-axis indicates the direction of laser scanning.

**Figure 2:**
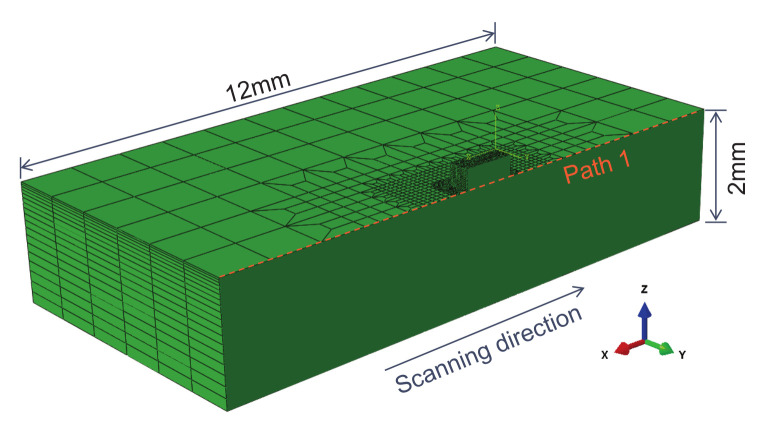
The finite element model contains two parts: the powder layer and the substrate. The dimension of the substrate is 12 × 10 × 2 mm, and the area of the powder layer is 1.2 × 0.6 mm.

Figure 2 shows the established finite element model, which contains two parts: the powder layer and the substrate. The dimension of the substrate is 12 × 10 × 2 mm, and the area of the powder layer is 1.2 × 0.6 mm, and the powder layer contains nine layers of the powder, and the thickness of each layer is 0.03 mm.

Calculations are done over half of the geometry taking advantage of symmetry. The laser beam travels along the positive *x*-axis. The positive *z*-axis represents the build direction vertically upward. The boundary conditions for the Abaqus-based mechanical analysis include a fixed bottom surface to ensure no movement, and the displacements of all nodes of the bottom surface along the *x*, *y* and *z* directions are zero.

The additive manufacturing process parameters are summarized in Table 1. Based on the trial calculation results, the calculation step time of the laser scanning process for each layer is 1/50 of the scanning time, and the calculation step time of the powder spreading process for each layer is 1/10 of the powder spreading time.

### 2.2. Transient Heat Transfer Analysis

When the laser heat source irradiates the surface of the powder layer, only part of the laser energy is absorbed and the rest is reflected. The absorbed laser energy melts the metal powder to form a small pool. When the molten pool solidifies, a metallurgical bond is formed between the adjacent laser scanning track and the adjacent powder layer. During this process, besides heat conduction between materials, heat convection and heat radiation are also considered.

The governing equation of three-dimensional transient heat conduction can be expressed as,

(2) $$\rho c_{p}\frac{\partial T}{\partial t}=\frac{\partial }{\partial x}\left(k_{x}\frac{\partial T}{\partial x}\right)+\frac{\partial }{\partial y}\left(k_{y}\frac{\partial T}{\partial y}\right)+\frac{\partial }{\partial z}\left(k_{z}\frac{\partial T}{\partial z}\right)+Q$$

where ρ represents the material density, $c_{p}$ is the specific heat capacity, *k* is the coefficient of heat conduction, and *Q* denotes the intensity of the heat source.

Furthermore, the natural boundary conditions can be represented as,

(3) $$k\frac{\partial T}{\partial n}-q+q_{conv}+q_{rad}=0{}^{}\left(x,y,z\right)\in S$$

where *S* represents the surface subjected to heat flux, convection and radiation, and *n* is the normal vector of surface *S*, *q* is the heat flux, and $q_{conv}$ represents the surface convection, $q_{rad}$ is the thermal radiation, and,

(4) $$\begin{array}{c} q_{\mathrm{conv}\;}=h\left(T-T_{\infty }\right) \end{array}$$

(5) $$\begin{array}{c} q_{\mathrm{rad}\;}=\epsilon K_{b}\left[{\left(T-T_{z}\right)}^{4}-{\left(T_{\infty }-T_{z}\right)}^{4}\right] \end{array}$$

where *h* is the convective heat transfer coefficient, $T_{\infty }$ denotes the ambient temperature, ϵ is the emissivity of the materials, $K_{b}$ is the Stefan–Boltzmann constant and $K_{b}$ = 5.67 × 10${}^{-8}$ W/m${}^{2}$K${}^{4}$, and $T_{z}$ is the zero absolute temperature.

### 2.3. Thermo-Mechanical Model

To determine the residual stress of the materials during the SLM process, a thermo-mechanical model is proposed. Based on the incremental theory of plasticity, the total strain increment $\varepsilon^{\mathrm{tot}}$ consists of several effects under small strain regime assumption, as,

(6) $$\varepsilon^{\mathrm{tot}}=\varepsilon^{e}+\varepsilon^{p}+\varepsilon^{\mathrm{th}}$$

where $\varepsilon^{e}$ and $\varepsilon^{p}$ are the elastic, plastic strain components, respectively. $\varepsilon^{\mathrm{th}}$ denotes the thermal strain. Then the elastic strain increment can be expressed as,

(7) $$\varepsilon^{e}=D^{-1}\sigma ,\mathrm{and}\;D=\frac{\nu }{E}+\frac{1+\nu }{E}I$$

where σ is the stress, *E* and ν are the elastic modulus and Poisson’s ratio, respectively; I is the unit vector. while the plastic strain as:

(8) $$\varepsilon^{p}=\lambda \sigma^{\mathrm{dev}},\mathrm{and}\;\sigma^{\mathrm{dev}}=\sigma -\frac{1}{3}tr\left(\sigma \right)1$$

where λ denotes the plastic flow factor, and

(9) $$\begin{array}{ccc} \lambda =0 & \;\mathrm{if}\; & \sigma_{\mathrm{vm}}<\sigma_{p}\\ \lambda >0 & \;\mathrm{if}\; & \sigma_{\mathrm{vm}}>\sigma_{p} \end{array}$$

here $\sigma_{p}$ represents the yield stress, $\sigma_{\mathrm{vm}}$ denotes the effective von Mises stress:

(10) $$\sigma_{\mathrm{vm}}=\sqrt{\frac{3}{2}{\left(\sigma^{\mathrm{dev}}\right)}^{T}\sigma^{\mathrm{dev}}}$$

Based on the von Mises yield criterion, the flow stress and plastic strain development in a single-pass multilayer SLM process are simulated using a temperature-dependent plastic model.

The thermal strain increment can be represented as:

(11) $$\varepsilon^{\mathrm{th}}=\alpha \Delta T$$

where α is the coefficient of thermal expansion (CTE), ΔT shows the temperature increment.

### 2.4. Material Properties

During the SLM processing, the initial state of the materials is a powder state, which will melt instantly after the high-energy heat source of the laser beam. When the laser beam is far away, the molten pool rapidly solidifies into a solid. The thermal and mechanical properties of the material also change nonlinearly during the phase transition with temperature. The thermal and mechanical properties of materials exhibit great influence on the accuracy of temperature field and stress field simulation in the SLM process. The thermal physical parameters involved in the calculation of temperature field mainly include thermal conductivity, specific heat capacity and density, while the mechanical parameters involved in the calculation of stress field mainly include elastic modulus, tangent modulus, yield strength, Poisson’s ratio and thermal expansion coefficient.

**Figure 3:**
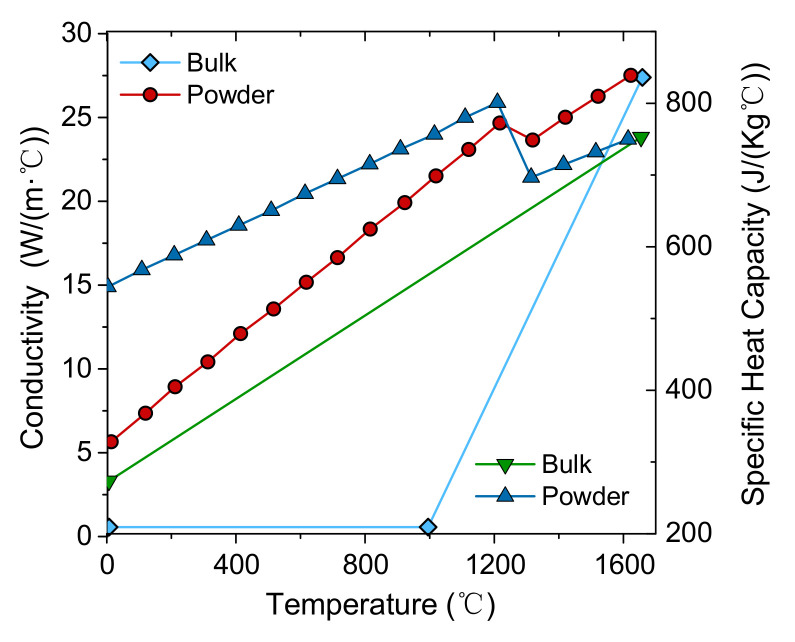
Thermal conductivity and specific heat capacity in bulk and powder state of Ti-6Al-4V.

The material properties related to temperature for the Ti-6Al-4V alloy investigated in the present work are depicted in Table 2, which are provided by Refs. [20,27]. Moreover, the temperature-dependent thermal conductivity and specific heat capacity in bulk and powder state of Ti-6Al-4V alloy are shown in Figure 3.

In the SLM process, materials often exist in three states: powder state, liquid state and solid state. The material is controlled by temperature and state changes occur due to the limitation of curing temperature and liquefaction temperature. In the present work, the relationship between the states of the materials and the temperature is established by using a USDFLD user-subroutine in the FE solver Abaqus/Standard.

## 3. SLM Process Predictive Simulations

At present, the numerical simulation methods to solve the temperature field, deformation/thermal stress distribution and flow field of the SLM process can be divided into two categories [28]: the finite element method and the natural strain-based method. The basic principle of the finite element methods is that the heat source is simulated by heat flux on the grid surface covered by the heat source, and the temperature field distribution is obtained by solving the heat conduction equation. The natural strain-based method is taking the natural strain as the source of internal stress, and the deformation of the whole structure is solved by a purely elastic finite element analysis. However, the current simulation methods show limitations when applied to the SLM process. Although the simulation of the SLM process based on the finite element method has high precision, the mesh size must be smaller than the thickness of the powder layer, which causes high computational cost. While the algorithm based on natural strain needs to build a huge natural strain library for finite element analysis.

**Figure 4:**
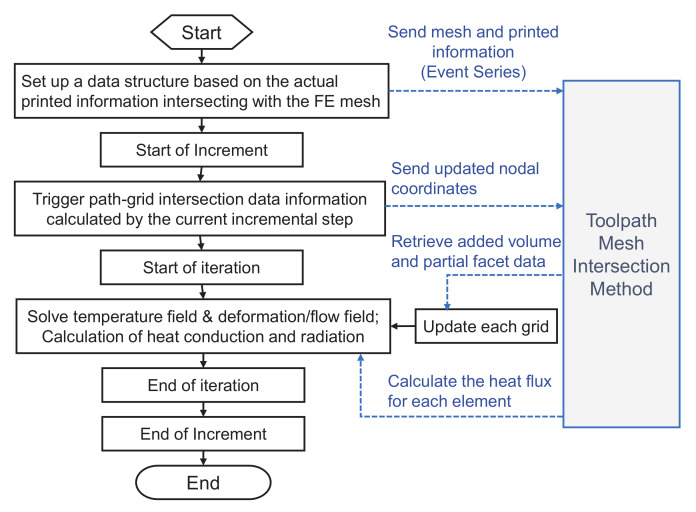
Schematic diagram of the toolpath-mesh intersection Method in SLM processing.

To accurately and efficiently simulate the SLM manufacturing process, a toolpath-mesh intersection method is used in the present work [20,22]. The schematic diagram of the toolpath-mesh intersection Method is demonstrated in Figure 4, which can be divided into four steps:

(i) Begin calculation, the actual printed information and the finite element mesh intersecting data (event series) are sent to path-mesh intersecting data module.
(ii) For each incremental step, the path-grid intersection information (the volume added to the grid, the location of the laser scan) is triggered during the printing time calculated by the current incremental step.
(iii) Update each grid based on the information triggered by the current incremental step.
(iv) The temperature field is solved, and the heat conduction and radiation are calculated based on the activated surface of the grid.

## 4. Results and Discussions

### 4.1. Temperature Distributions and Molten Pool Configurations

The temperature of the SLM process has an important influence on the final microstructure and mechanical properties of structures. Therefore, it is necessary to generate the evolution of the temperature field during the SLM process, so as to provide a theoretical basis for process optimization and obtaining excellent quality parts.

**Figure 5:**
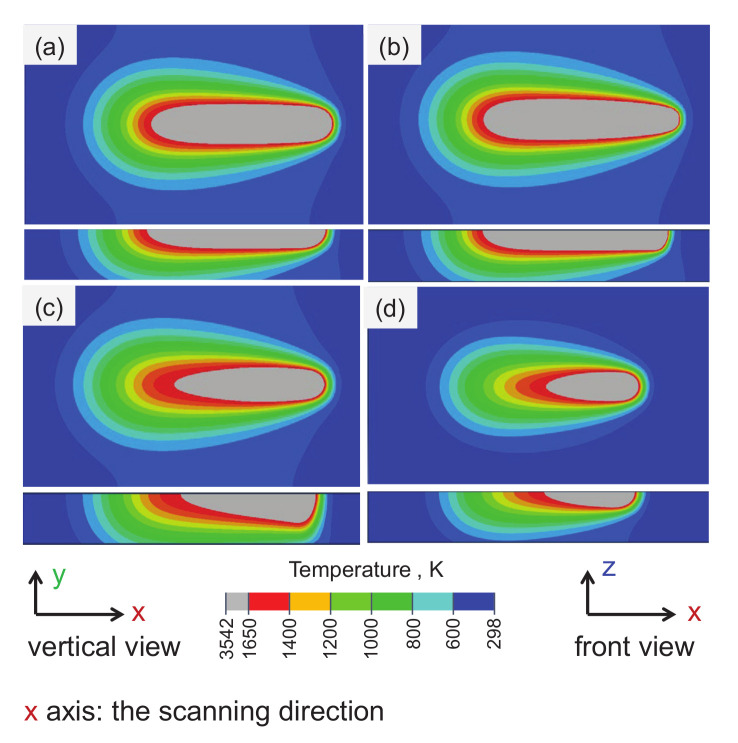
The transient temperature distribution on the top surface (top view) and lengthwise section (front view) of the molten pool during the SLM process with different heat source power and parameters. The heat source parameters c=2a, $f_{f/r}$ = 1, (**a**) *a* = 0.05 mm, b=0.06 mm, *P* = 190 W; (**b**) *a* = 0.025 mm, b=0.06 mm, *P* = 190 W; (**c**) *a* = 0.05 mm, b=0.15 mm, *P* = 190 W; (**d**) *a* = 0.05 mm, b=0.06 mm, *P* = 100 W.

Figure 5 shows the transient temperature distribution on the top surface (top view) and lengthwise section (front view) of the molten pool during the SLM process with different heat source power and parameters. The gray area indicates that the temperature field is greater than 1650 ${}^{\circ }$C at the current time, which is higher than the melting temperature of Ti-6Al-4V alloys, and can be regarded as the molten pool area. When the laser reaches the center of the first powder layer, the temperature field isotherms on the upper surface of the pool are similar to a series of ellipses, and the isotherms at the front of the pool are denser than those at the back, which show the same trend as that in literature [29]. The main reason may be that the thermal conductivity of the material increases during the transformation from powder state to solid state, resulting in the powder layer heat transmission. Furthermore, in laser processing, the energy lost through heat conduction is usually higher than that lost through thermal convection/radiation. With the gradual accumulation of the scanning layer, the heat dissipation through heat conduction is gradually weakened, which further reduces the loss of laser energy and also causes the higher temperature at the higher powder layer and larger molten pool.

Figure 5a,b shows that when parameter *a* decreases from 0.05 mm to 0.025 mm, the energy of the Goldak heat source model is concentrated, and the predicted maximum temperature of the molten pool increased from 3540 ${}^{\circ }$C to 5885 ${}^{\circ }$C, while the width of the molten pool decreases from 128 μm to 100 μm. The results show that *a* has little effect on the prediction result of molten pool depth. Figure 5a,c show that when parameter *b* increases from 60 μm to 150 μm, the predicted depth of molten pool is no longer uniform, and the maximum depth of the molten pool changes from 60 μm to 90 μm, the predicted maximum temperature in molten pool decreased from 3540 ${}^{\circ }$C to 2970 ${}^{\circ }$C, and the width of the molten pool also decreases from 128 μm to 100 μm.

#### Effects of Laser Power

Figure 5a,d illustrate the effects of the laser power on the morphology of the molten pool. As the laser power decreases from 190 W to 100 W, the size of the molten pool decreases significantly: the width of the molten pool decreases from 128 μm to 90 μm, and the depth decreases from 60 μm to 45 μm, the predicted maximum temperature in the molten pool decreases from 3540 ${}^{\circ }$C to 3048 ${}^{\circ }$C. Furthermore, the heat-affected zone of the molten pool under larger laser power is significantly larger than that under smaller laser power.

**Figure 6:**
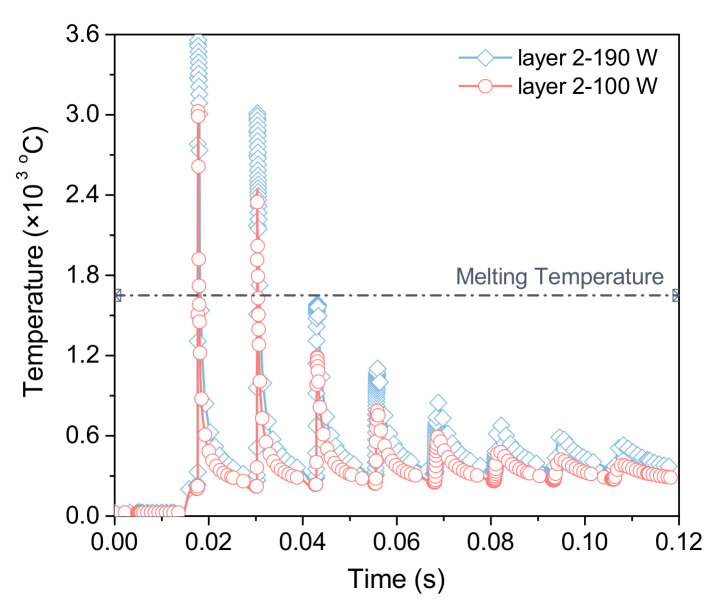
Temperature evolution at the center of the 2nd layer under different power conditions.

Figure 6 demonstrates the temperature measured at the center of the 2nd layer with the laser scanning time at different laser powers. The curves fluctuate significantly with the laser scanning time, and each wave represents the completion of a laser scan, the slope of the curve represents the cooling rate. When the laser approaches the center point, the temperature increases rapidly. As the laser moves away from the point, its temperature drops promptly, resulting in a high cooling rate. Additionally, under the two power conditions, the first two peaks of the temperature curves are greater than 1650 ${}^{\circ }$C, that is, both power conditions can penetrate the current scanning layer and remelt the next layer. Meanwhile, attributing to the fact that the heat accumulation effect and the cooling time of each layer are similar in the SLM process, the average temperature at the same position increases gradually after each layer is printed, which means that the heat stored in the previous layer influences the next processing layer.

**Figure 7:**
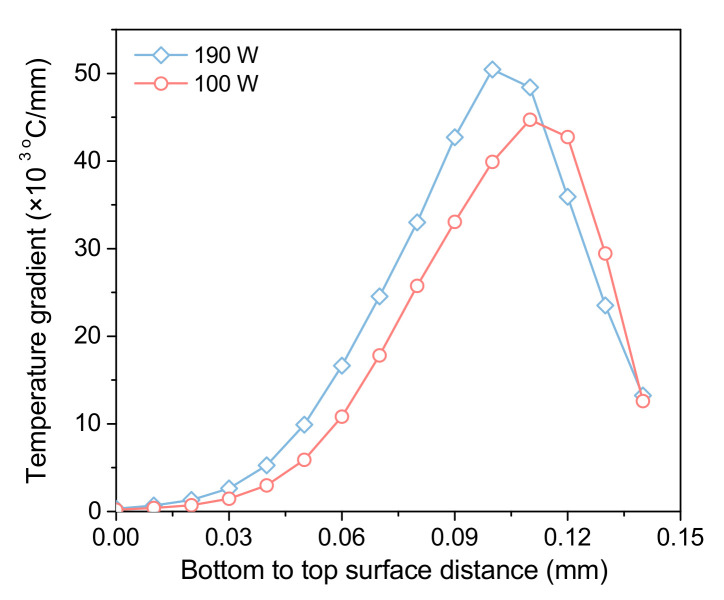
Temperature gradient changes from the bottom to the molten pool surface in the printing depth direction.

Figure 7 displays the temperature gradient in the printing depth direction of the molten pool. Because the solidified material has greater thermal conductivity than the liquid material, gradually reducing the temperature gradient along with the depth of the molten pool, the temperature gradient decreases gradually from the top surface to the bottom of the molten pool.

Moreover, the cooling rate of the molten pool increases with increasing laser power. It must be pointed out that a larger cooling rate means that larger residual stress may be generated in the forming process. The molten pool formed under low power has a low temperature and short existence time, which reduces the fluidity of the molten pool and may produce pores in the forming process. Therefore, appropriate process parameters must be considered based on the thermal history in the actual forming process.

### 4.2. Residual Stresses Analysis

#### 4.2.1. Solidification Process

In the process of laser scanning, the distribution of temperature field is nonuniform, resulting in the temperature gradient and thermal stress. When the laser irradiates the powder layer, the local temperature of the powder layer increases with the input of high energy density, and then decreases rapidly with the departure of the laser heat source. Plastic deformation occurs when the thermal stress is higher than the yield limit of the material. When the laser scanning is finished, the temperature of the materials drops to room temperature, and the internal stress is redistributed: the so-called residual stress.

**Figure 8:**
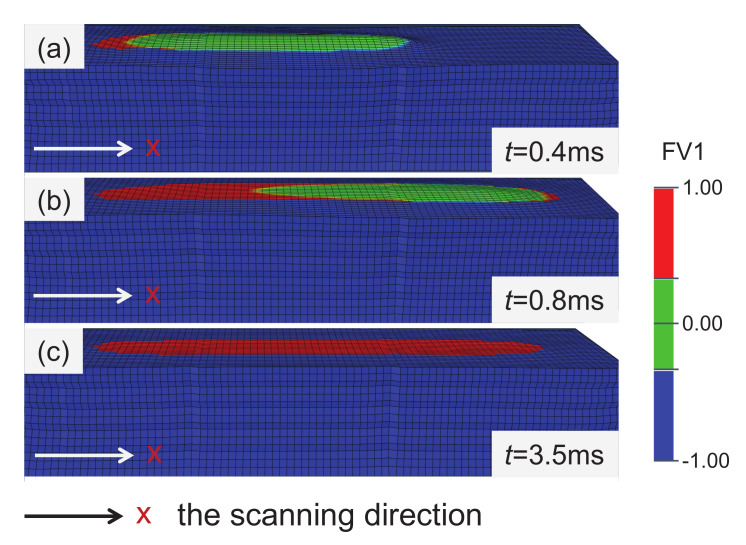
The melting solidification process of Ti-6Al-4V alloy at different times. Green represents the liquid area and red represents the solid area, deformation is magnified by 10 times. (**a**) *t* = 0.4 ms; (**b**) *t* = 0.8 ms, and (**c**) *t* = 3.5 ms.

The melting solidification process of the material in the SLM process is shown in Figure 8, and the cloud diagram is the result after the deformation is magnified by 10 times. The powder melts when the temperature increase under laser irradiation. Then, the material becomes liquid phase and expands, and its height is higher than that of the powder layer. When the laser is far away, the material temperature decreases and finally solidifies. The material shrinks during the cooling process, and its shrinkage is constrained by the surrounding colder materials, resulting in tensile stress, which has significant effects on the mechanical properties of the products [30,31].

#### 4.2.2. Residual Stresses Analysis

The stresses along *x*, *y* and *z* directions are referred to as the longitudinal, transverse and through-thickness stresses, respectively. From the calculated stress field, these individual residual stress components are extracted along paths 1, and the path 1 has been defined in Figure 2 by a dashed red line. The longitudinal residual stress along path 1 is important because it is a driving force for crack propagation, buckling and distortion.

**Figure 9:**
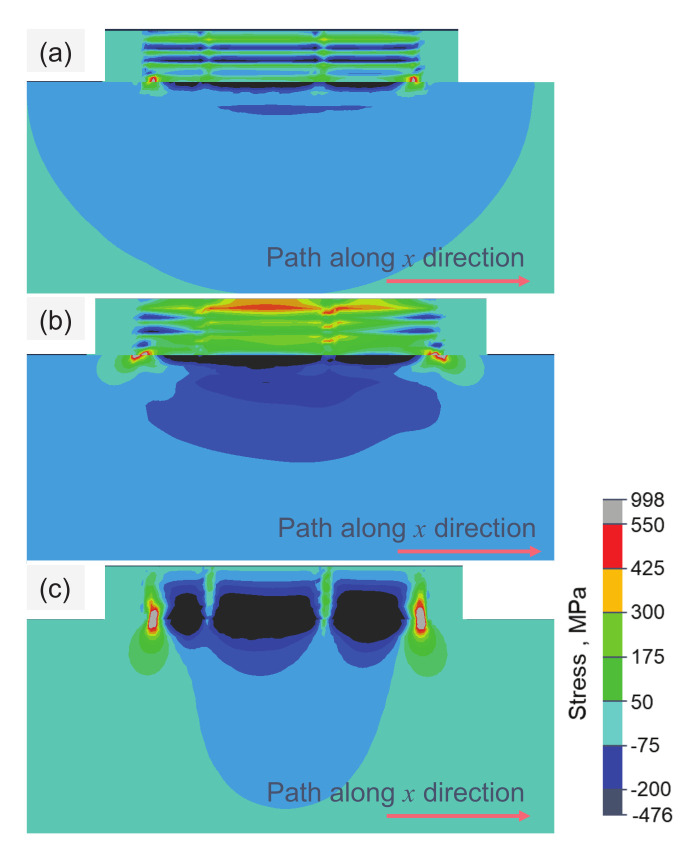
Longitudinal strain field at the end of depositing. (**a**) $\sigma_{11}$, (**b**) $\sigma_{22}$ and (**c**) $\sigma_{33}$ in the 4th layer of Ti6Al4V powder on substrate. Laser beam scanning direction is along the positive *x*-axis.

Figure 9 shows the residual stress distributions of $\sigma_{11}$, $\sigma_{22}$ and $\sigma_{33}$ at the end of the 4th layer printing, and $\sigma_{11}$, $\sigma_{22}$ and $\sigma_{33}$ represent the stresses along *x*, *y* and *z* directions, referred to as the longitudinal, transverse and through-thickness stresses. The location of the maximum tensile residual stresses of $\sigma_{11}$ and $\sigma_{22}$ appear near the top layer after the printing of the 4th layer, which has the same trend as the results in Ref. [15].

Furthermore, the residual stress changes from tension to compression at the interface of both ends of two continuous layers, and there is a steep stress gradient. This result shows that 3D printing parts are prone to bending or warping at the edge. As shown in Figure 9c, $\sigma_{33}$ is in a compressed state at the center of the printing part, while it is in a stretched state near the interface between the deposited layer and the substrate. In extreme cases, it may lead to the delamination, separation or even warping of the printing part from the substrate.

**Figure 10:**
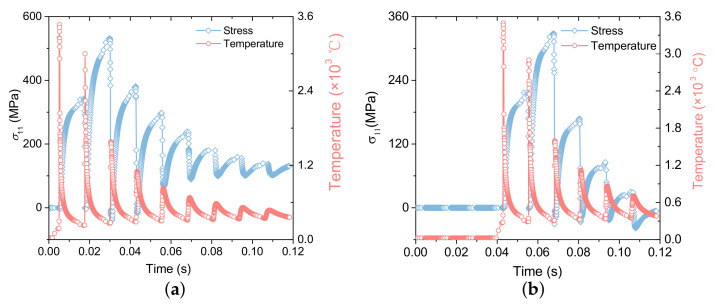
Stress and temperature distribution path along *x*-direction at the end time of each layer. (**a**) Stress and temperature distribution path along *x*-direction of the 1st layer. (**b**) Stress and temperature distribution path along *x*-direction of the 4th layer.

The changes of $\sigma_{11}$ at the center points of the 1st and 4th layers are illustrated in Figure 10. Before the temperature reaches the peak for the first time, the center point of the layer is in the state of powder or melting, and its stress state is zero; Then the temperature at this point began to decrease. When it was lower than the solidification temperature (1604 ${}^{\circ }$C), tensile stress occurs in the material, and the stress gradually increased with the distance from the heat source. When the temperature reaches the peak for the second time and is higher than the melting temperature, the stress at this point is rapidly released to zero; Then as the temperature decreases, the tensile stress is generated again at this point.

It should be noted the tensile stress peak generated in the second time of each layer is greater than that in the first time. The possible reason is that the current layer and the next layer can be melted during laser scanning, and after each layer is scanned, the temperature of the next layer is lower than that of the top layer, that is, during solidification, the next layer will produce a larger temperature gradient. Therefore, the residual stress generated by the next layer is greater than that generated by the top layer. Moreover, when the temperature at the central point starts to rise for the third time, certain compressive stress will be generated, as the temperature peak for the third time is less than the melting temperature. During SLM processing, the temperature of the material changes periodically with the scanning time, resulting in the phenomenon of ’thermal expansion and cold contraction’ of the material. When the material is heated and expanded, it is constrained by the extrusion of the surrounding materials in the *x* direction. Then the temperature decreases again, and the tensile stress is generated again at this point, resulting in the stress of the material changing with the scanning time.

**Figure 11:**
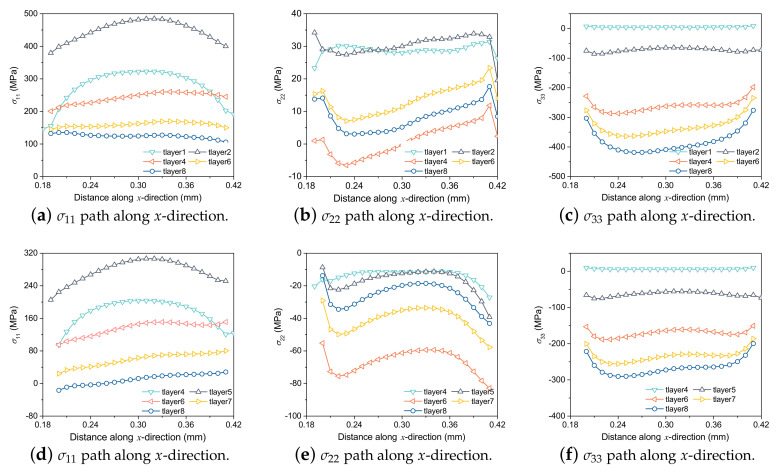
Residual stress distributions of (**a**–**c**) the 1st layer and (**d**–**f**) the 4th layer at the end time of each layer.

Figure 11 illustrates $\sigma_{11}$, $\sigma_{22}$ and $\sigma_{33}$ of the center line along the printing direction after the printing of the 1st and 4th layers, respectively. With the increase of printing layers, the material is continuously reheated and cooled, and $\sigma_{11}$ is gradually released and reduced layer by layer. However, after two more printing layers are added, the current layer will no longer be melted, and the compressive stress on the material will gradually accumulate, so $\sigma_{33}$ increases layer by layer. While $\sigma_{22}$ is not affected by the number of printing layers.

**Figure 12:**
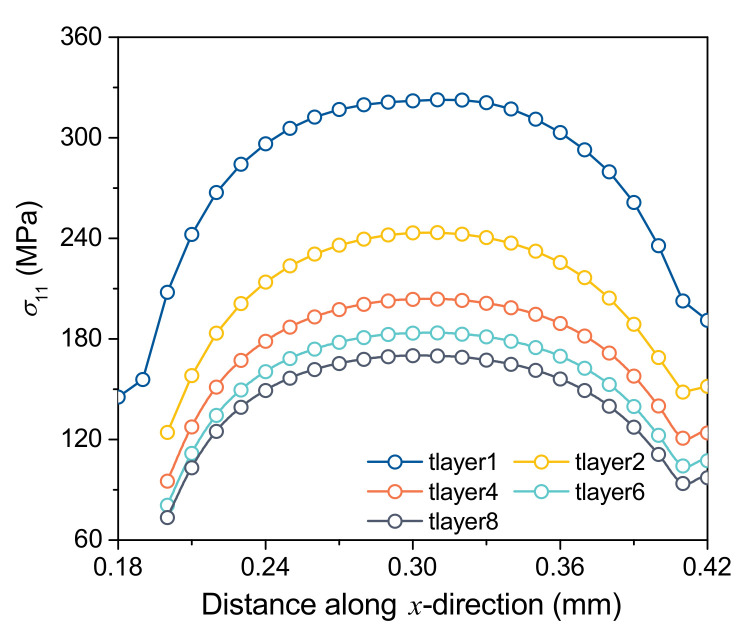
The position along the center line of the printing direction, and the $\sigma_{11}$ distribution of the top layer after the printing of each layer.

Furthermore, the distributions of $\sigma_{11}$ on the top layer after the printing of each layer are displayed in Figure 12. Due to the heat accumulation effect in the printing process, the temperature of the top layer increases layer by layer after the printing of each layer, and the temperature gradient in the solidification process decreases layer by layer. Therefore, $\sigma_{11}$ of the top layer decreases with the increase of the number of printing layers.

**Figure 13:**
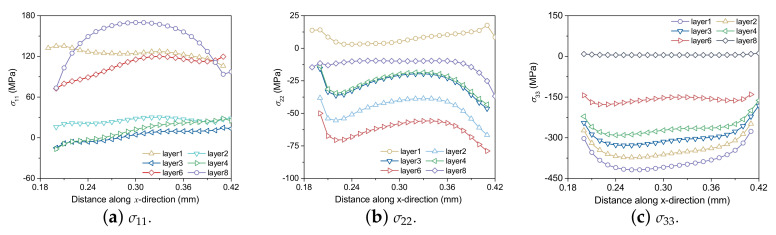
After printing of each layer, the distribution of (**a**) $\sigma_{11}$, (**b**) $\sigma_{22}$ and (**c**) $\sigma_{33}$. of each layer, layer *i* indicates the time when the printing of layer *i* ends.

Figure 13 demonstrates the distribution of $\sigma_{11}$, $\sigma_{22}$ and $\sigma_{33}$ of each layer after printing. It should be noted that the cooling time of 4.8 ms is increased after the printing is completed, then the $\sigma_{11}$ of the 1st layer in Figure 13a increases. The possible reason is that the $\sigma_{11}$ of the first layer is redistributed due to the temperature change of the substrate during cooling. This result shows that after the printing of parts is completed, the cooling rate of parts should be controlled by adjusting the temperature of inert gas, otherwise the risk of part cracking may be increased.

The simulation of the residual stress in SLM process can conduct printing path planning and control the residual stress generated in the printing process based on the simulation results, and carry out deformation compensation design to make the geometry of the printed structure conform to the theoretical model.

#### 4.2.3. Effects of Laser Power on Stress Field

**Figure 14:**
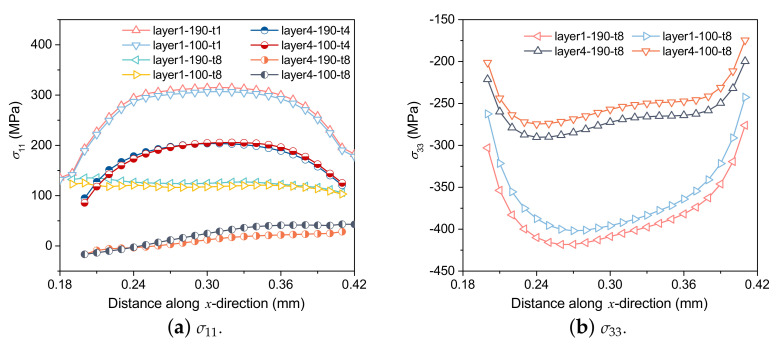
Comparison of stress distribution along the *x* direction at the center of the 4th and 8th layers under different laser power.

Figure 14 shows the effect of print power on stress distribution. The residual stress distribution is the same under different laser power conditions, with different residual stress values. With high laser power, the temperature gradient of parts is large, resulting in extreme residual stress [32]. However, the distribution of $\sigma_{11}$ under different laser power conditions seems to show little difference, which may be due to the fact that the scanning time of each layer (0.6 ms) is less than the cooling time (7.2 ms) after scanning of each layer. Due to the influence of cooling time, the difference in residual stress values under different laser power conditions is reduced.

#### 4.2.4. Sensitivity Analysis of Grid Size

The previous discussions are based on meso-scale model, which can reflect a detailed SLM physical process with a high computational cost. Moreover, the temperature in the molten pool changes violently, causing the calculation difficult to converge [33]. To simulate the SLM process of macroscopical parts, the fidelity of simulation can be controlled by selecting the appropriate calculation time increment and mesh size.

**Figure 15:**
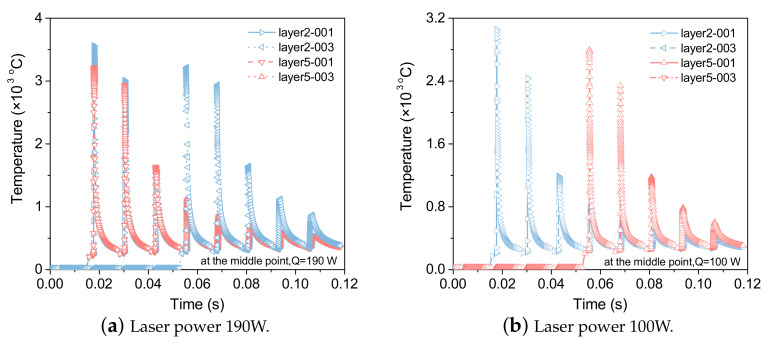
Temperature evolution at the center of the 2nd and 5th layers under different grid sizes.

The temperature change with printing time under different grid sizes and different laser power are shown in Figure 15. where 001 and 003 indicate that the grid sizes are 0.01 mm and 0.03 mm, respectively. Under the condition of a coarse grid, the result of the maximum temperature is slightly lower than that of a fine grid, and the other temperature calculation results are completely consistent. To accurately construct the morphology and temperature distribution of the molten pool in the calculation, a finer grid size is necessary.

**Figure 16:**
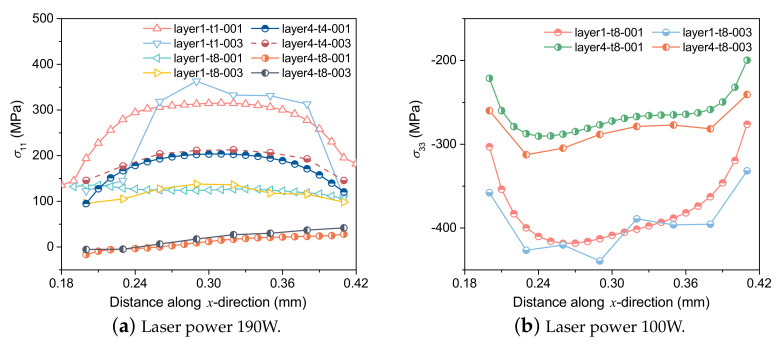
Stress distribution at the center of the 2nd and 5th layers under different grid sizes.

Furthermore, $\sigma_{11}$ and $\sigma_{33}$ of the 1st and 4th layer under different grid sizes with laser power 190 W are compared in Figure 16. When the grid size is represented by one layer of grid, the stress distribution results are similar to those under a fine grid.

In the SLM process, the area around the molten pool will rapidly generate high temperature gradients, resulting in anisotropic properties of the material. The current research ignores the performance differences of parts under different process parameters caused by the anisotropy of materials. To clarify the residual stress evolution in the process, the anisotropy-based model should be considered to simulate the mesoscopic stress field. Moreover, according to the results, the definition of “initial temperature”, grid size and incremental step of calculation time have a great influence on the simulation results. Based on the current process conditions, the difference between simulation and actual results should be gradually calibrated, so as to realize the real application of simulation calculation in guiding the SLM production process.

## 5. Conclusions

In the present work, a general framework for Ti-6Al-4V alloys in the SLM process is established based on the toolpath-mesh intersection method, which can predict temperature evolutions, molten pool configurations and residual stresses. The effects of the laser power and grid size on the temperature distributions and residual stresses are details discussed. Additionally, the solid-state phase transformation for materials in the SLM process are well simulated, which can be used to explain the complex physical phenomena inside molten pool phenomenologically. The main conclusions are as follows:

(1) Due to the rapid solidification of melted powders, the residual stress along the laser scanning path produces a large stress gradient at the edge of the parts, which may lead to the maximum deformation along the printing direction appearing at the edge of the parts, and cause cracks.
(2) The maximum tensile residual stress in the plane appears near the top layer, and decreases layer by layer with the increasing number of printing layers. At the interface between two continuous layers, the residual stress changes from tension to compression. The stress concentration occurred at the interface between the melt layer and the substrate may lead to warping deformation and even cracking.
(3) The stress parallel to the scanning direction is the main stress causing cracks in SLM parts, as the stress parallel to the scanning direction is larger than the stress perpendicular to the scanning direction.
(4) The toolpath-mesh intersection method can effectively predict the SLM process, but the grid size and incremental step of computing time have a great impact on the simulation results, and further improve the prediction accuracy of the SLM process is the direction that needs to be further investigated.

## Figures and Tables

**Table 1:** Process parameters for the simulation of SLM process.

Process Parameters	Value	Unit or Notes
Power of heat source, *P*	190/100	W
Scanning speed, *v*	1000	mm/s
Scanning direction along *x* axis	0.6	mm
Printing time for each layer	4.8 $\times \;10^{-3}$	*s*
Cooling time for each layer	7.2 $\times \;10^{-3}$	*s*
Layer thickness, *h*	0.03	mm
Ambient/powder temperature, $T_{\infty }$	26	${}^{\circ }$C
Substrate temperature, $T_{textB}$	200	${}^{\circ }$C

**Table 2:** Latent heat of Ti-6Al-4V [20,27].

Parameters	Value	Unit
Latent heat of fusion	2.86 × 10${}^{5}$	J/kg
Solidus temperature	1604	${}^{\circ }$C
Liquidus temperature	1650	${}^{\circ }$C
Latent heat of vaporization	9.83 × 10${}^{6}$	J/kg
Liquidus temperature	3290	${}^{\circ }$C
Vaporized temperature	3390	${}^{\circ }$C
Density	4420	kg/m${}^{3}$
Emissivity	0.25	∼
Convection Coefficient	18	W/(m${}^{2}\cdot$${}^{\circ }$C)
Laser absorptivity	50	%

## Data Availability

Not applicable.
